# Professional nurses’ perspectives on the influence of Ideal Clinic status on patient care quality in eThekwini, South Africa

**DOI:** 10.4102/hsag.v30i0.3064

**Published:** 2025-12-15

**Authors:** Ntombifuthi N. Mazibuko, Thembelihle S.P. Ngxongo, Dudu G. Sokhela

**Affiliations:** 1 Department of Nursing, Faculty of Health Sciences, Durban University of Technology, Durban, South Africa

**Keywords:** ideal clinic status, facility streams, healthcare providers, healthcare users, quality of care

## Abstract

**Background:**

As the global community commits to achieving universal health coverage by 2030, countries such as South Africa also acknowledge that delivering optimal healthcare that would universally cover all populations is multifactorial and includes the existence of infrastructure, medical supplies, healthcare providers and quality improvement initiatives. Such initiatives should focus on providing comprehensive, effective, safe and people-centred care that would address the healthcare needs of the population at an affordable cost. The Ideal Clinic initiative is the latest quality improvement strategy the South African government implemented, aiming to improve quality healthcare services at the primary care level. Studies on Ideal Clinic indicate that even though primary healthcare (PHC) performance on standards was achieved, quality clinical services remained a challenge, hence the need for further research.

**Aim:**

This study aimed to explore the professional nurses’ (PNs) perspectives on the influence of ‘Ideal Clinic’ status on quality patient care in PHC facilities.

**Setting:**

The study was conducted in nine PHC clinics that were purposively selected from on district in KwaZulu-Natal.

**Methods:**

An exploratory qualitative research design using semi-structured interviews with eight purposively sampled PNs.

**Results:**

Staff shortages, a lack of specialised training, inadequate physical space, shortage of medicine and essential stationery compromised the quality of care in PHC facilities.

**Conclusion:**

The Ideal Clinic status did not equate to quality of care and healthcare services should be monitored and evaluated based on the streams within facilities where clinical care begins and quality is established.

**Contribution:**

Understanding and acknowledging PNs’ views regarding healthcare service provision and the need to assess quality healthcare in each facility stream.

## Introduction

### Background

Primary care is the first level of healthcare that covers a comprehensive range of preventive, promotive, curative and rehabilitative health services. Up referral to secondary and tertiary levels of care or down referral provides the possibility for continuity of healthcare for the population (Haque et al. 2020; Mulenga 2025). Health services at the primary care level focus on disease preventive strategies that are aimed at eradicating, eliminating and minimising the impact of disease or, if none of these are feasible, retarding the progress of disease and disability (Wendimagegn & Bezuidenhout 2019). In South Africa, primary healthcare (PHC) is freely available to all populations regardless of their socio-economic status. As a result, health for all is enshrined in several chapters of the South African Constitution (*Act 108 of 1996*) as a basic human right. The constitution further emphasises the decentralisation of healthcare service delivery to Districts, hence ‘the District Health System’, which is the vehicle and organisational framework for delivering PHC in South Africa (Fusheini & Eyles 2016). Primary healthcare is nurse-driven, and contracted or full-time employed medical practitioners only visit the PHC facilities on specific days to consult with healthcare users (HCUs) who have complicated health conditions that require specialised medical care. In most PHC facilities, HCUs’ access to specialised healthcare services by adjunct healthcare professionals, which includes dental care, was made possible through a referral system, which progressed from lower to higher levels of care. Matolengwe, Murray and Okafor (2024) believe that referral systems are intended to maximise HCUs’ access to quality comprehensive healthcare, while preventing unnecessary overcrowding and waste of specialised healthcare resources. Despite the positive increase in public utilisation of primary care services, most PHC facilities are not suitable to cater to the increased number of HCUs. Subsequently, health system challenges have arisen, which include staff shortages, congestion, negative staff attitudes, long waiting times and inadequate medical supplies, which compromise safe and quality healthcare (Hunter et al. 2017; Malakoane et al. 2020).

The South African National Department of Health (NDoH) has introduced several quality improvement initiatives to enhance healthcare efficiency, safety and the quality of service delivery and access for all HCUs at the primary level (Maphumulo & Bhengu 2019). The ‘Ideal Clinic’ initiative is the most recent effort focused on improving and maintaining the quality of the services provided (Hunter et al. 2017; South Africa, NDoH 2020). An ‘Ideal Clinic’ is defined as a facility with good infrastructure, covering physical condition and space, availability of essential equipment, information and communication tools, adequate staff, medicines and supplies, efficient administrative processes and sufficient bulk supplies. Such a facility applies relevant clinical policies, protocols and guidelines, supported by partners and stakeholders, to deliver quality and comprehensive healthcare services to the community (South Africa, NDoH 2020). The ‘Ideal Clinic’ has 10 components, namely: administration, integrated clinical services management, pharmaceuticals and laboratory, human resources, support services, infrastructure and bulk supplies, health information management, communication, district health system support, and implementing partners and key stakeholders.

The ‘Ideal Clinic’ was later called Ideal Clinic Realisation and Maintenance (ICRM), aiming at improving and maintaining quality healthcare through revitalising and preserving the quality of infrastructure, human resources, good governance and equipment for PHC facilities to achieve positive health outcomes for the populations (Massyn, Pillay & Padarath 2019).

The ‘Office of Health Standard Compliance’ (OHSC) determines the ‘Ideal Clinic’ status through comprehensive quality assessments and monitoring of compliance with healthcare delivery standards. The OHSC employs 33 sub-components and 238 elements to evaluate facility performance. Elements are weighted as non-negotiable vital elements, where failure to meet them could result in loss of life; vital elements that directly impact service delivery and clinical care of HCUs; essential elements related to processes and structures that indirectly influence the quality and safety of clinical care; and important elements that affect the healthcare environment (South Africa, NDoH 2020). Each element is scored based on facility performance, and the Ideal status is awarded to facilities achieving 100% in all non-negotiable vital elements, 60% – ≥ 80% in vital elements, and 50% – ≥ 70% in essential elements. Responsibility for achieving these standards is shared among the PHC facility, Health District, the Provincial authorities and NDoH. The awarded status can be Platinum, Gold or Silver, with Silver being the lowest score. Platinum status is bestowed upon the top-performing PHC facilities delivering the highest quality healthcare to their populations compared to those with Silver status.

Ideal clinic realisation and maintenance had a positive impact on improving quality care for HCUs, with 56% of facilities being ideal. However, ongoing maintenance and improvement of the ideal status remained a challenge (Muthathi & Rispel 2020). Inadequate physical space in facilities in relation to the population being served leads to poor adherence to infection control protocols, possible drug stockouts because of the inadequate storage capacity of bulk supplies, cleanliness issues, as well as long waiting times for the HCUs. Staffing levels and allocation also depend on the availability of physical space at the facility. Although the introduction of the Ideal Clinic initiative had a positive impact on clinical quality indicators and generated improvements in standards adherence by PHC facilities, changes implied by improved facilities’ performance on quality standards did not automatically translate to improved healthcare services. There was minimal impact on the actual improvement of quality clinical services provision measures and output. Challenges, such as inadequate medical supplies and medicine stock-outs, staff shortages and cleanliness of PHC facilities still exist. These challenges were among the six priority areas that the South African NDoH identified for fast-track as early as 2011 and were regarded as fundamental to the provision of safe and decent care (Muthathi & Rispel 2020; South Africa, NDoH 2018; Stacey et al. 2021). The PHC services are predominantly nurse-driven, with Professional Nurses (PNs) constituting the majority of healthcare providers in PHC facilities. As such, PNs are both legally and morally accountable for delivering quality healthcare services. Consequently, the researcher sought to explore PNs’ perspectives on how the Ideal Clinic status influences the quality of patient care in PHC facilities.

### Problem statement

Studies show that the provision of quality services in public health facilities still faces challenges such as long waiting times and insufficient space to attend to HCUs effectively, which has led to negative experiences. Despite the effort and interventions put in place by the NDoH through the introduction of the Ideal Clinic initiative to correct deficiencies in the quality of PHC services, quality of care is hindered by overwhelming staff workload, a lack of environmental cleanliness, poor infrastructure and a lack of medical equipment and consumables (Matlala et al. 2021).

One in two individuals (48.7%) with hypertension were unscreened and undiagnosed, and 23% were screened, but the results were not interpreted. The HCUs with diabetes were insufficiently screened for complications and co-morbidities because of a lack of medical equipment (Shisana et al. 2019). Poor quality service provision, especially at the PHC level, results in a loss of confidence by HCUs, which escalates medico-legal claims, burdening both health services and professionals irrespective of the 2013 Ideal Clinic initiative to improve quality of care (Bresick, Von Pressentin & Mash 2019).

Although the eThekwini District is well-endowed with a high number of PHC facilities, there is a slow achievement of critical health outcomes and sub-optimal HCU satisfaction with the quality of care that undermines such investment (eThekwini District Health Plan [DHP] 2018/19–2020/21). The study intends to assess if the Ideal Clinic status translates to an improved quality of clinical service provision for HCUs at the PHC level in terms of availability of physical space in the facility, cleanliness, waiting times, functional medical equipment and availability of medicines.

### Purpose of the study

The study aimed to explore and describe the PNs’ views regarding the influence of ideal clinic status on the quality of patient care provision in PHC facilities.

### Research objective

The objective of this study was to explore the PNs’ perspectives on the influence of Ideal Clinic status and patient care quality.

## Research methods and design

### Study design

The qualitative, exploratory descriptive design was used to explore the perspectives of PNs on the influence of Ideal Clinic status on the quality of patient care in PHC facilities in eThekwini District, KwaZulu-Natal. In this design, narratives from the study participants are analysed to find meaning and to provide a description of the experience that promotes deeper understanding (Gray & Grove 2021). The influence of Ideal Clinic status on the quality of patient care was explored using semi-structured interviews with eight purposively sampled PNs from nine selected PHC facilities.

### Study setting

The study was conducted in the eThekwini District in KwaZulu-Natal, South Africa. EThekwini is one of the 11 health districts, a large Metropolitan area located in the coastal region of KwaZulu-Natal (EThekwini DPH 2018/2019–2020/2021). The fixed PHC facilities were identified as key study foci, in which the findings would be generalised and were therefore used as study sites. Facilities were distributed across the three sub-districts of eThekwini metropolitan and were coded as N, S and W to ensure anonymity. The nine PHC facilities were managed by both the eThekwini Municipality and the Provincial Department of Health; however, they were all audited and awarded with Ideal Clinic status by the OHSC. The ideal status was either Platinum, Gold or Silver.

### Population and sampling

The population was PNs working in PHC facilities with ideal status. Purposive sampling was used to select nine fixed PHC facilities, three from each sub-district, as follows: two from Authority A and one from Authority B, as well as eight PNs who worked in acute, chronic and maternal, child and women’s health because of the valuable information they had that benefited the study. Box 1 depicts the number of participants in all sub-districts. The sample size for study sites and PNs was guided by data saturation as described by Guest, Namely and Chen (2020).

BOX 1 Study participants from eThekwini’s three sub-districts.

**Table:** 

Authorities	Authority A				Authority B				Grand total
Sub-districts	N	S	W	Total	N	S	W	Total	8
Participants	2	1	2	5	1	1	1	3	Participants

N, North sub-district; S, South sub-district; W, West sub-district.

#### Inclusion criteria

The PNs who worked across the selected PHC facilities for 2 years or more were also purposively sampled.

#### Exclusion criteria

Health posts, mobile clinics, community healthcare centres and gateway clinics were excluded because they were not targeted in the study. The PNs who worked in PHC facilities for less than 2 years or were employed by non-governmental organisations.

### Data collection tool

An interview guide with one broad question and a few pre-determined probing questions was utilised to collect data from participants as indicated in Table 1. Semi-structured interviews were conducted and audio recordings were used to record participants’ comments. Field notes were also written to substantiate data collected and to capture non-verbal cues during interviews. The interview guide was developed in English because the researcher who conducted the interviews and the interviewees were proficient in English. Data collection tools were pilot-tested before conducting the research.

**TABLE 1:** Questions from interview guide.

Broad question	Probing questions
What influence does Ideal clinic status have on quality of healthcare service provision in PHC facilities?	What can you say about the quality of services that you provide in this facility?
	Can you describe the support system that you get in working at the Ideal Clinic?
	What challenges are you experiencing in working at the Ideal Clinic?
	What would be your recommendations regarding solutions to identified challenges?

PHC, primary healthcare.

### Data collection

Data were collected from March to June 2024 after approval from the Institutional Research Ethics Committee permission obtained from eThekwini Municipality Research Committee and the KwaZulu-Natal Provincial Department of Health. After the study was explained, written consent was obtained from each participant who met the inclusion criteria for the study before the interviews.

Semi-structured interviews were conducted in English using a pre-tested interview guide with pre-determined open-ended and probing questions. To maintain confidentiality and privacy, the interviews were held in an unused consulting room. Each interview lasted about 45 min and was audio-recorded with permission from the participants to provide an accurate record of the participants’ comments during data analysis. The field notes were also written to substantiate and capture the non-verbal cues of participants. Data saturation was monitored during data collection at each facility before proceeding to the next facility across all sub-districts. Saturation was reached when themes and categories became repetitive and redundant after six interviews conducted across the sub-districts. To confirm data saturation, two more interviews were conducted, totalling eight interviews.

### Data analysis

Data were analysed concurrently with data collection using thematic analysis. The purpose was to organise, provide structure, and elicit meaning from the research data (Polit & Beck 2021). Data were transcribed from the audio recorder and field notes into a written format. The transcribed interviews were captured into a master file through Microsoft Word. Eight steps of Tesch’s open coding approach were used for data analysis (Tashakkori, Johnson & Teddlie 2021; Tesch 2013). In steps 1 and 2, the transcripts were repeatedly read, the recordings were listened to, and field notes were reviewed to gain an overall sense of the data, establish the necessary background, and understand the emotions and experiences of participants. The thoughts that emerged from the data were written down, known as familiarisation and immersion.

In steps 3 and 4, the information gathered from the interviews was organised into a list of topics that were either major, unique or leftover. Related topics were grouped as categories and sub-categories, in which themes and sub-themes emerged in step 6. In steps 7 and 8, sub-themes were identified from existing themes. Findings were presented under each theme and supported by verbatim statements to ensure the authenticity of the findings.

### Trustworthiness

Trustworthiness refers to the integrity of the researcher, appropriateness of research methods and the legitimacy of the results (Rose & Johnson 2020). To ensure trustworthiness, the study adhered to the principles of credibility, confirmability, transferability, dependability and authenticity as described by Lincoln and Guba (1985).

### Credibility

Credibility refers to confidence in the truth of the data and their interpretation by the researcher. It is achieved when the research findings provide an accurate understanding of data that are derived from participants’ true perceptions (Rose & Johnson 2020). To ensure credibility, the same standard broad question and pre-determined probing questions were used across all study participants until data saturation was reached. Transcripts were repeatedly read and compared with the audio-recorded information and the field notes for the overall interpretation of the findings. The transcribed data and field notes will be kept for 5 years for a record trail.

### Transferability

Transferability refers to the probability that the study findings have meaning for others in similar situations and can be applied to other settings or groups (Polit & Beck 2021). Transferability was ensured by providing a thick description of the entire research process, which included data collection, analysis and the context of data collection to facilitate the process of future researchers, who might endeavour to conduct a similar study in other settings.

### Dependability

Dependability can be established by ensuring the reliability of the data through varied periods and conditions (Lincoln & Guba 1985). It also concerns documentation of steps taken and decisions made during data analysis. Although the participants were interviewed on different dates, the interview guides used for all participants contained the same standard, broad questions and pre-determined probing questions to ensure dependability. The data collection tools were also piloted before data collection.

### Confirmability

Confirmability is concerned with the objectivity, accuracy and relevance of data. The purpose is to illustrate clearly the evidence and thought processes that led to the conclusions (Rose & Johnson 2020). Confirmability was achieved by developing and maintaining a trail of data records that described the entire research process. All interview transcripts and audio records were kept safe and only accessed by the researcher. The excerpts and direct quotes from data were used to support the themes that emerged during data analysis.

### Ethical considerations

The study followed the basic ethical principles of autonomy, beneficence and justice described by Polit and Beck (2021). The study received full ethics approval from the eThekwini Municipality Research Committee and the KwaZulu-Natal Provincial Department of Health (NHRD Ref: KZ_202304_015), gatekeeper permissions from the KwaZulu-Natal Provincial Department of Health Research Unit, eThekwini Municipality Head of Health Unit and from eThekwini Health District. Permission to access facilities was sought from the PHC facility managers using email. To observe the principle of respect for persons, participants were given full written information about the study in a language they could understand. Study participants were allowed to exercise self-determination, and informed written consent was obtained from each participant as described by Gray and Grove (2021). Participants were informed that they could cease participation in the study at any stage if uncomfortable. Confidentiality was ensured through coding of participants and study sites; electronic data were stored in a password-protected computer only known to the researcher and hard copies of data were kept under lock and key and information was only used for research purposes.

## Results

### Demographic data

Eight purposively selected participants, of whom five (62.5%) were from facilities under authority A and three (37.5%) from authority B. This discrepancy in the number of participants from the two health authorities was because five PHC facilities included in the study were from health authority A and three from authority B, of the 60:40 ratio of the distribution of the PHC facilities between the two health authorities in the eThekwini district (EThekwini DHP 2018/19). Representation of both study sites and participants was distributed across the three sub-districts as indicated in Box 1. The eight participants were Africans, of whom two were men. The level of experience working at a PHC setting for seven participants ranged from 2 years – 10 years and one participant had 25 years’ experience (Table 2).

**TABLE 2:** Abbreviations used in participants’ quotes.

Abbreviations	Meaning
BN1: HCP1: F38	BN1= Managing Authority B, N= North sub-district, 1= Facility 1: HCP1= Healthcare Provider 1: F38= Female, age 38.
AN1: HCP1: F36	AN1= Managing Authority A, N= North sub-district, 1= Facility 1: HCP1= Healthcare Provider 1: F36= Female, age 36.
BW1: HCP1: M33	BW1= Managing Authority B, W= West sub-district, 1= Facility 1: HCP1= Healthcare Provider 1: M33= Male, age 33.
AS1: HCP1: F29	AS1= Managing Authority A, S= South sub-district, 1= Facility 1: HCP1= Healthcare Provider 1: F39= Female, age 29.
AS2: HCP1: F45	AS2= Managing Authority A, S= South sub-district, 1= Facility 2: HCP1= healthcare Provider 1: F45= Female, age 45.
AW2: HCP1: F38	AW2= Managing authority A, West sub-district, 1= Facility 2: HCP1= Healthcare Provider 1: F38= Female, age 38.
BS1: HCP1: M46	BS1= Managing Authority B, S= South sub-district, 1= Facility 1: HCP1= Healthcare Provider 1: M46= Male, age 46.
BN1: HCP2: F49	BN1= Managing Authority B, N= North sub-district, 1= Facility 1: HCP2= Healthcare Provider 2: F49= Female, age 49.
AW1: HCP1: F52	AW1= Managing authority A, W= West sub-district, 1= Facility 1: HCP1= Healthcare Provider 1: F52= Female, age 52.

## Findings and discussions

After data analysis, three main themes were identified, namely staff establishment, physical space in PHC facilities, facility organisation and patient flow. Furthermore, six sub-themes emerged from the identified themes, as indicated in Table 3.

**TABLE 3:** Themes and sub-themes of the study.

Theme	Sub-theme
1. Staff establishment	1.1 Staff training and support 1.2 Staff allocation 1.3 Patient workload
2. Physical space in primary healthcare facilities	2.1 Inadequate medical supplies and equipment 2.2 Shortage of medicines
3. Facility organisation and patient flow	3.1 Clinical records and replacements

### Theme 1: Staff establishment

The participants verbalised that the quality of healthcare services provision in PHC facilities is dependent on the number of staff allocated and available in each PHC facility. The emphasis was that services at the PHC level were nurse-driven; therefore, the staff establishment needed to include all staff categories, like enrolled nurses and nurse assistants, clerks and medical doctors. According to participants, formal or informal staff training on primary care courses and support would result in improved healthcare service provision. Participants agreed that they were sometimes updated on new clinical guidelines and protocols that enhanced the provision of efficient and safe care to HCUs. However, the emphasis was on the need for formal training. The participants had the following to say regarding staff skills and training:

‘… our clinic is like a hidden community health centre because we are open 24 hours and even do deliveries, yet the medication, equipment, and staff are for a small clinic. We don’t even have a clerk at night to make or pull out files for patients, hence you see most of our patients are carrying duplicate papers for us to use as cards … clinic cards are misfiled and there is no continuity of care.’ (BN1: HCP1: F38)

‘… I wish I could be formally trained in primary health care. I am allocated to see sick people, yet I am only trained in family planning … It’s hard because I am not sure if I am doing the right thing for patients.’ (AN1: HCP1: F36)

‘… I am working in the acute stream, yet I don’t have any additional training to help me consult with patients … I requested to work with pregnant women, but the manager told me that staff have to rotate … to tell the truth, I am not sure what I am doing for patients.’ (BW1: HCP1: M33)

Participants continued to raise concerns about staff allocation that was carried out on a rotational basis across all three facility streams without considering their skills, training or experience, which negatively affected staff performance and the quality of services rendered to HCUs. Participants highlighted that excessive patient workloads, both in terms of high patient volumes and complex cases, were further exacerbated by expectations to carry out tasks normally performed by lower-level staff, contributing to increased stress and potential impacts on care quality. Participants had the following comments:

‘I end up listening to patients’ problems and just giving medication that I think will help the patient … I know that is not legally correct, but I do it to finish all patients allocated to me because most of the time I am working alone.’ (AS1: HCP1: F29)

‘Consulting with first-visit pregnant women takes a very long time because of the procedures that are supposed to be done to them. I wish they were not combined with sick children because IMCI assessment also takes long … this is time-consuming and tiring, especially if it is done by one person.’ (AS2: HCP1: F45)

‘I feel they need to give us enough staff so that we see a reasonable number of patients per nurse. For now, I don’t think our staffing levels are not in relation to our facility status, which is Platinum.’ (AW2: HCP1: F38)

‘[*T*]o work a little faster, I end up just dispensing medication for the patients with files that are kept at the clinic and doing documentation later … I know that is not legally correct, but I do it to finish all patients allocated to me; however, I do forget sometimes to record.’ (BS1: HCP1: M46)

### Theme 2: Physical space in primary healthcare facilities

Participants raised concerns about the limited physical space in most PHC facilities, which could not allow separate facility streams. In such facilities, HCUs shared waiting areas and Healthcare Providers (HCPs) shared consultation rooms, which compromised patients’ privacy. Each facility had one central point where medical supplies were kept for the entire facility and stocks were ordered based on the availability of storage space. As a result, there was a shortage of medical supplies, such as gloves, masks, bandages and cotton wool, syringes, needles and hand paper towels. This was evident in the following comments:

‘We are expected to do pap smears on most female patients, even if there are no gloves in the facility, because each professional nurse has a target number of pap smears to be done in a month. We are all affected … yesterday our enrolled nurse had to do wound dressing of a patient with a septic caesarian section … she covered her hands with specimen plastics …’ (AS2: HCP1: F45)

Concerns were further raised in terms of the shortage of essential medical equipment. Participants verbalised that they were sharing equipment among themselves, especially in facilities where movable medical equipment was used. The perspectives of the participants regarding the availability of medical supplies and equipment were expressed by the following experts:

‘… can you imagine, every time I need to examine the patient, I have to move around to borrow an auroscope from another room … I think the manager hides these instruments because she gives us when there are auditors …’ (BN1: HCP2: F49)

‘… sometimes we do get Ideal Clinic status, but I wonder how because we don’t have so many things here … we only see some medical equipment during audits and after that, we don’t have. I am tempted to say the medical equipment is borrowed elsewhere just for audit purposes but not for patient care … I think such acts mask the actual situation we are facing at this clinic.’ (AN1: HCP1: F36)

Participants indicated that medicine storage rooms were inadequate and sometimes were without air conditioners to regulate room temperatures. They further verbalised that medicines were ordered from the main pharmacy every month, basing the amount of orders on the availability of storage space for medicines like vaccines and for chronic conditions, disregarding medicine usage. Most of the items in the order form were reported to be out of stock or to follow, meaning there would be no medicines until the next order or delivery. That was evidenced in the following excerpts:

‘… even if we want to stock more medicines, there is not enough storage space in our medicine room. The patients don’t understand that and think we don’t order enough medicines from the main pharmacy. Medicines finish before the delivery of the next order … the pharmacist from the main pharmacy limits the number of emergency orders that can be processed while waiting for the main order … this leaves us without stock for the patients.’ (BN1: HCP2: F49)

‘When receiving the main order, half of the items that we order are out of stock … indicated as o/s meaning “out of stock” or s or f meaning “stock to follow.” If our manager follows up with the pharmacy, there is nothing that gets delivered, but instead, the pharmacist will ask for an emergency order where we are supposed to put only limited urgent stock.’ (AW1: HCP1: F52).

### Theme 3: Facility organisation and patient flow

Space constraints in most facilities prevented the reorganisation of facilities into acute, chronic and mother, child and women’s health streams as specified by Ideal Clinic prescripts. Participants verbalised that even floor signs that were supposed to direct patients to different streams were fading or even blocked in congested facilities, resulting in HCUs following the wrong queues. Furthermore, most participants verbalised that security guards were utilised as queue marshals to direct patient flow instead of ensuring safety in facilities. This was evidenced by following comments:

‘… when you say Ideal Clinic, the clinic space is supposed to be divided to suit patient flow, but here, as you can see, the physical structure of the clinic does not allow it. As a result, the patients end up joining the wrong queues.’ (AW1: HCP1: F52)

‘… just look at our clinic infrastructure; it is so limiting, even if we want to separate our patients, it’s impossible … I wish the clinic infrastructure could be improved or give us park homes as additional structures to extend our clinic.’ (AS2: HCP1: F45)

Participants from most facilities verbalised that there was a shortage of facility-based clinical records for HCUs with acute conditions, resulting in the use of halved A4 pieces of paper for documenting patient information. Participants further indicated that files for HCUs with acute conditions were either filed or misfiled, resulting in difficult retrieval. Essential stationery, like the Road to Health booklets for children, was inadequate, which made replacements of lost booklets impossible. As a result, documentation and record-keeping of clinical information were compromised. The views of participants were expressed in the following excerpts:

‘… most of the time, the clerk only files back chronic patients’ records and clinical records for patients with acute problems, which get dumped in a box because the filing room and cabinets are not enough for all files … The load of files is huge and the clerk cannot retrieve some files … it becomes so difficult for me to do follow-up care in acute patients because most of the time, they are given blank duplicate papers to write on.’ (BN1: HCP2: F49)

## Discussion

The study provided insights into the lived experiences of PNs working at PHC facilities regarding the influence of Ideal Clinic status on the quality of patient care provision in PHC facilities that were awarded either silver, gold or platinum status in eThekwini Metropolitan Municipality, KwaZulu-Natal province, South Africa. The findings revealed that PNs who worked in PHC facilities with Ideal Clinic status were experiencing several challenges that interfered with the quality of healthcare service provision. The challenges included staff shortages, inadequate training and support, skewed staff allocation in terms of their skills, excessive patient caseload, insufficient medical supplies and equipment, shortages of medicines and essential stationery. These findings aligned with those of Nesengani, Downing and Ten Ham-Baloyi (2025), who state that the heavy workload experienced by PNs is a significant barrier in delivering safe and quality care. The Ideal Clinic initiative aimed to revitalise and correct all the challenges of service provision in PHC facilities. The overall goal was to systematically transform all PHC facilities to meet national health standards to achieve the government’s mission of improving the population’s health status by providing quality healthcare (South African NDoH 2020).

### Staff establishment

An Ideal Clinic initiative requires that all categories of staff be available in each stream for effective service provision. Interviews revealed that staffing levels in the facilities did not align with the number of HCUs served. The volume of HCUs exceeded the available number of PNs, and the complexity of patients’ health problems contributed to an unmanageable caseload. Ideal Clinic prescripts specify that staff allocation should be calculated using workload indicators of staffing needs, which would ensure a balanced nurse-patient ratio (South Africa, NDoH 2020). Participants verbalised that because of work overload, in trying to finish the crowd before the end of the day, they sometimes resorted to listening to the HCUs’ main complaints and dispensing medication without performing examinations. Other authors had similar findings in that high HCU load in healthcare facilities resulted in PNs prioritising documentation of clinical findings and disregarding communicating them to HCUs, thus compromising the safety of care (Joseph et al. 2022).

Most PNs received support and training through short courses by the Department of Health, thus enabling them to function effectively in PHC facilities. However, concerns were raised about the slow pace in training on the specialised Primary Care course, which would equip PNs with more skills for consulting HCUs. Gabrani, Schindler and Wyss (2020) state that empowering HCPs with skills and competence plays a key role in providing quality services.

Documentation of clinical notes was completed later, when all HCUs were gone, especially for those HCUs with facility-based records, which led to some PNs not recording clinical notes or recording only what they could remember about the consultation process. The study findings concur with those by Bresick et al. (2019), who found that healthcare systems in Africa are under-resourced in terms of human resources and PHC services delivered by low-level and poorly trained HCPs, which compromises the quality of healthcare received by HCUs. Muthathi and Rispel (2020) attest that the increased PHC services utilisation rate resulted in staff overload and poor healthcare services, including HCUs not being examined and some special tests, like pap smears, being postponed.

The participants were also concerned about inadequate staff training and support. Although the PNs were allocated based on their clinical skills, most of them did not have specialised training in Clinical Nursing Science, Health Assessment, Treatment and Care, a specialised course that is prescribed by the South African Nursing Council for all PNs at the PHC level (*Nursing Act 2005*). Allocating PNs on a rotational basis from one stream to another worsened the situation where skills alignment was not per PNs’ duties, which compromised the safety of HCUs. Gabrani et al. (2020), in their study, suggested that healthcare services should be provided by skilled and competent PNs to minimise medical and preventable errors.

### Physical space in primary healthcare facilities

Integrated clinical services management in the Ideal Clinic initiative advocates that each facility stream should have its waiting area, observation, storage room, file and fully furnished consultation rooms and dedicated PNs. In most selected facilities, there was one central point where all HCUs were admitted, observation was performedand thereafter directed to different facility streams where feasible; otherwise, the HCUs waited in the main reception area for a consultation. The findings showed that some facilities did not have storage rooms, and supplies were ordered according to the availability of space, which resulted in medical supply stock-outs and shortages. The challenges in accessing important supplies such as gloves, masks, bandages or toilet paper from the main stores impacted the performance of procedures such as pap smears. In most facilities, it was reported that medical equipment such as an ophthalmoscope, thermometers, haemoglobin and glucometers and stethoscopes was either shared among PNs or not available. As a result, in some instances, PNs had to omit the examination of HCUs if the equipment was still in use by another PN. Such acts compromised the timeliness, efficiency and effectiveness of care rendered to HCUs. However, the PNs indicated that a surplus of supplies and medical equipment was noticed when facilities had to be audited. Similar findings were reported by Khan, Albobali and Kamal (2022), who found that 61% of PNs reported a shortage of medical equipment in facilities, and the maintenance plan of such equipment was not monitored, resulting in inadequate functional equipment to enhance the provision of quality healthcare services. Also, the findings by Muthathi and Rispel (2020) revealed that some PHC facility managers resorted to paying from their own pockets to buy equipment or borrow from other facilities just to comply with the Ideal Clinic audit to get an ideal status. Such acts resulted in ‘fake compliance’ and inappropriate Ideal Clinic status being awarded to facilities that were still experiencing challenges in providing quality healthcare services. Muthathi, Levin and Rispel (2019) also state that facility managers do not have the power to influence procurement processes and turnaround times for facility stock orders. As a result, facilities have to wait for the supply chain department to deliver supplies, which affects service provision in terms of timeliness and effectiveness of care.

In most facilities, medicines were ordered from the main pharmacy based on facility usage and in accordance with designated order days outlined in the Ideal Clinic prescripts. However, some participants were concerned that the availability of medicine storage space was a major factor in deciding on the amount of medicine that was ordered. As a result, medicine order levels were based on the availability of storage space. The PNs felt that the situation was worsened by medicine stock-outs from the main pharmacy, resulting in most facilities having medicine shortages, which compromised the effectiveness and efficiency of healthcare provision. The study findings were similar to those by Motsepe, Wolvaardt and Webb (2020), who concluded that medicine supply shortages threatened healthcare efficiency and the health of the public by creating barriers to optimal care. These authors further state that shortages of essential medicines have been reported globally, owing to their high costs. Stacey et al. (2021) in their study found that improved satisfaction with quality standards in ideal facilities was driven by the support and resources provided to such facilities and not solely by the introduction of quality standards themselves. Therefore, support to PHC facilities in terms of all required resources is vital for improved quality healthcare provision.

### Facility organisation and patient flow

The Ideal Clinic initiative requires PHC facilities to be reorganised into designated, fully furnished and adequately staffed streams for acute, chronic and maternal, child and women’s health (MCWH) services, each supported by relevant facility-based clinical records (South Africa, NDoH 2020). The HCUs living with chronic illnesses had facility-based clinical records, while some HCUs with acute illnesses used either hand-held or facility-based records. However, most HCUs that came for acute problems had hand-held clinical records. Shortage of essential stationery, which included maternity records, Road to Health Booklets and facility-based records for HCUs with acute conditions, was a concern for most participants. As a result, photocopied pages or small carrier cards were used to record clinical notes of HCUs, which compromised recording with possible medical litigations and the safety of HCUs. In facilities with adequate facility-based clinical records, storage space was a concern. Clinical records were either kept in cabinets in the reception areas, separate park homes or kept in cupboard boxes on the floor. In most cases, the clerks could not file or retrieve such clinical records. Where files could not be retrieved, HCUs were provided with empty duplicate cards to proceed to the consultation rooms. As a result, effectiveness, efficiency and continuity of healthcare were compromised. The study’s findings concur with those by Matlala et al. (2021) and Makau and Khunou (2022), which indicate that insufficient physical space and congestion in health facilities contributed to long waiting hours, delays in accessing services and negative experiences for HCUs. Shisana et al. (2019) believe that increased medical malpractice litigations and claims are attributable to poor or no documentation on HCUs’ clinical records, thus preventing the South African Department of Health from contesting the claims from HCUs because of inadequate or the absence of clinical information on HCUs’ clinical records. Keeping clear, accurate and eligible clinical records is integral to the delivery of quality healthcare services as it forms the basis of safe, transparent and measurable clinical practice (Khan et al. 2022). Participants’ views indicated that there were still major challenges in PHC facilities that were awarded Ideal Clinic status.

### Limitations of the study

The Ideal Clinic initiative is a South African healthcare quality improvement strategy for PHC facilities. However, the study was conducted in one district (eThekwini) in KwaZulu-Natal and included only nine PHC facilities. The study only included PNs; however, PNs in PHC facilities comprise multidisciplinary members who also provide healthcare services. Out of the 10 components of the Ideal Clinic initiative, the study only concentrated on part of sixth component, which is infrastructure. The remaining 9 components of the Ideal Clinic could also contribute to both negative and positive findings of the current study.

### Recommendations

Based on the findings of the study, the researcher makes the following recommendations.

The policymakers should review the processes, procedures and audit tools for Ideal Clinic status accreditation to include perceptions of PNs regarding the quality of the clinical services being offered, and the perceptions of HCUs of clinical service quality in PHC facilities, if improved quality healthcare is to be patient-centred.

Institutional management monitors through audits, the consultation process, documentation keeping and retrieval of clinical records as part of Ideal Clinic audits. This will help capture the reality of the state of quality of services provided at PHC facilities before awarding the Ideal Clinic status.

## Conclusion

Several healthcare system-related factors have been, and still are, influencing the quality of service provision at PHC facilities in South Africa, such as staff establishment and physical space in facilities. The credit that some authors have attributed to the gains made by the Ideal Clinic initiative in improving quality healthcare service provision cannot be discounted; however, quality of care in middle-income countries such as South Africa has been declining as indicated by negative patient experiences in healthcare facilities. The factors influencing the quality of care in South Africa include inadequate infrastructure, a component of the ideal clinic initiative that involves all the non-negotiable vital elements, the loss of which in PHC facilities could result in the loss of HCUs’ lives or prolonged recovery. The NDoH promotes the implementation of ICRM in all PHC facilities as a quality improvement initiative to attain and sustain Ideal Clinic status as a way of improving quality healthcare. Much effort has been put in place in terms of policies and guidelines to aid quality service provision; however, these study findings indicate that the South African health system is still facing challenges to poor quality of PHC services in the form of inappropriate and inadequate infrastructure, inadequate medical equipment, inadequate supply of essential medicines and other consumables and negative staff attitudes.
